# Anaplastic Solitary plasmacytoma of mandible, masquerading as sarcoma

**DOI:** 10.12669/pjms.293.3406

**Published:** 2013

**Authors:** Mohamad Javad Ashraf, Negar Azarpira, Bijan Khademi

**Affiliations:** 1 Mohamad Javad Ashraf, MD, Department of Pathology, Shiraz University of Medical Science, Shiraz, Iran.; 2 Negar Azarpira, MD, Transplant Research Center, Shiraz University of Medical Science, Shiraz, Iran.; 3 Bijan Khademi, MD, Department of Otolaryngology, Shiraz University of Medical Science, Shiraz, Iran.

**Keywords:** Plasmacytoma, Mandible, Radiology

## Abstract

Plasma cell neoplasm is characterized by a monoclonal neoplastic proliferation of plasma cells and solitary plasmocytoma of bone (SPB) is a localized form. It usually occurs in vertebrae and secondarily in long bones. Its presence in mandible is extremely rare event. A 48-year-old man consulted to our clinic with a chief complaint of pain in his mandible. Radiography revealed a destructive lesion in body and ramus. The initial pathologic evaluation revealed a high grade pleomorphic neoplasm. The diagnosis was confirmed by immunohistochemical markers. Overall, plasmacytoma with anaplastic features can be confused with high grade sarcoma clinically and histologically**.**

## INTRODUCTION

Solitary plasmacytoma of the bone (SPB) is characterized by abnormal proliferation of malignant plasma cells, which usually occurs in axial skeleton, vertebrae and skull. Diagnostic criteria for SPB include a single lesion, negative skeletal survey, and tumor-free bone marrow. Patients are usually male and are in their sixth or seventh decade of life.^1^^,^^2^ The presence of SPB in jaws is extremely rare event, and the mandible is more frequently involved than the maxilla**. **Here we report a case of SPB of the mandible with destructive radiographic and anaplastic cytological findings.^2^^,^^3^

## CASE REPORT

**Fig. 1A, 1B:**
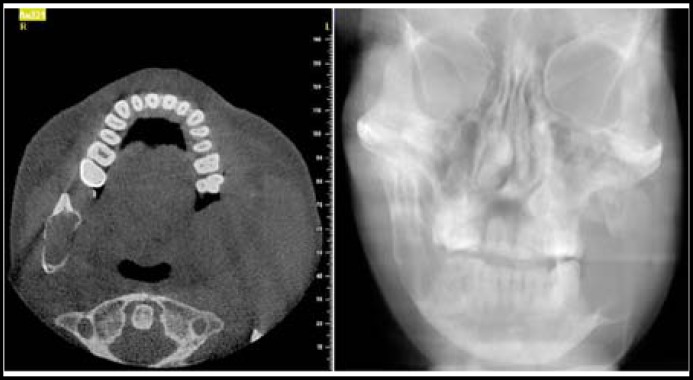
Computed tomography scan of mandible with complete destruction of ramus (Axial view and posteroanterior view).

**Fig.2:**
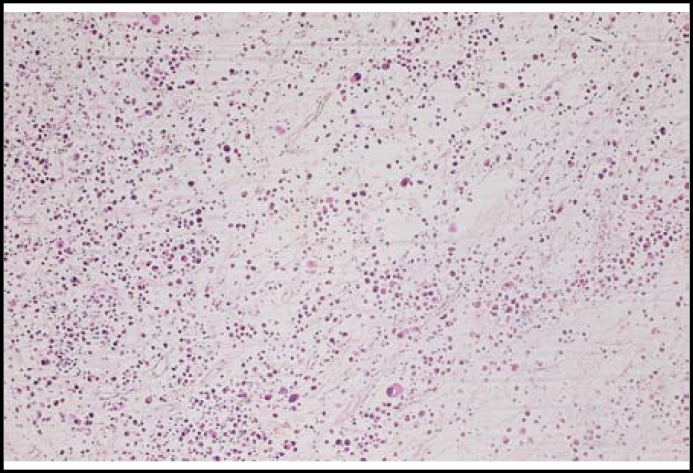
Sheet of Neoplastic plasma cells, showing eccentric nuclei and dense eosinophilic cytoplasm (H & E, ×400).

A 48-year-old man was referred to our laryngology clinic, complaining of pain in the left mandible and numb chin syndrome. The cone beam computed tomography (CBCT) revealed a large destructive lesion of the left mandible involving the body of the mandible and condylar processes (Fig. 1A and 1B). The skull and pelvis radiographic survey failed to identify additional osseous lesions. Complete blood count and coagulation tests were within normal limits. His past medical history was unremarkable. An incisional biopsy was performed under local anesthesia. Histopathologic examination of the specimen revealed proliferation of plasmacytoid cells with abundant eosinophilic cytoplasm, hyperchromatic nuclei, cellular pleomorphism with mitotic figures (Fig.2).

The differential diagnosis was plasma cell tumor and rhabdomyosarcoma. The malignant cells were immunopositive for kappa light chain and CD38 and negative for MyoD1, desmin, SMA, LCA and cytokeratin (DAKO, Denmark). The final diagnosis was solitary plasmacytoma of mandible. Boone marrow aspiration and biopsy revealed normocellular marrow with no evidence of involvement. The patient was referred to an oncologist and underwent local radiotherapy. No significant improvement in the condition of the patient was observed. Therefore, the patient underwent a left hemimandibulectomy. No evidence of recurrence was noted during a 9-month follow-up period. Written informed consent was obtained from patient and the study was approved by Research Ethics Committee of the Shiraz University.

## DISCUSSION

Plasma cell neoplasms are classified into three groups: multiple myeloma (MM), solitary plasmacytoma of bone (SPB), and extramedullary plasmacytoma.^1^ SPB accounts for 2% to 3% of all plasma tumors. Bataille and Sany critria,^1^ for SPB include an isolated tumor composed a clonal proliferation of plasma cells; absence of lesions on skeletal radiographic survey; absence of plasmacytosis in the bone marrow; and absence of anemia, Bence-Jones proteins in urine or hypercalcemia. Vertebrae and pelvic bones are the most common sites for SPB and jaw is rarely involved. The bone marrow–rich areas of jaw such as body, angle and ramus are the usual sites of involvement.^1^^,^^2^ Solitary plasmacytomas of other bones progress to MM in 35% to 75% of the cases in an average period of 38 months. It is not possible to predict which patients may develop systemic disease because no certain well known risk factor is identified. Therefore regular clinical follow up with monitoring of immunoglobulins and monoclonal proteins in serum and Bence-Jones proteins in urine is advised.^1^^,^^4^^,^^5^

The most frequent clinical symptoms of SPB are pain referred to the jaws and teeth, and less commonly pathologic fractures. Radiographically, the tumor appears as a unilocular or multilocular radiolucent destructive lesion and in our patient CBCT revealed a typical lytic radiolucent area.^2^^,^^6^

Plasma cells produce osteoclast-activating factors that promote the growth of osteoclasts and stimulate the bone resorption. Therefore, SPB usually appears on radiographic images as radiolucent areas.^7^^,^^8^

SBP is frequently misdiagnosed as benign lesions (ameloblastoma, odontogenic tumors), inflammatory disease such as osteomyelitis and granuloma and less frequently as malignant tumors (both primary and metastatic).^6^^,^^9^^,^^10^ Biopsy specimen analysis as well as bone marrow, serum protein electrophoresis with skeletal radiologic investigation confirms the diagnosis. Both surgery and radiation have been successfully used in the treatment of SPB.
